# Triple Therapy IDP-126 Gel for Acne Treatment: A Systematic Review and Meta-Analysis

**DOI:** 10.7759/cureus.74357

**Published:** 2024-11-24

**Authors:** Kennedy Sparling, Rakshita Giri, Anngela C Adams, Paul Kang, Victoria G Farley

**Affiliations:** 1 Medicine, University of Arizona College of Medicine - Phoenix, Phoenix, USA; 2 Medicine, Kirk Kerkorian School of Medicine, University of Nevada, Las Vegas, Las Vegas, USA; 3 Public Health, Mel and Enid Zuckerman College of Public Health, Phoenix, USA; 4 Dermatology, Vivida Dermatology, Las Vegas, USA

**Keywords:** acne vulgaris, adapalene, benzoyl peroxide, clindamycin, idp-126, meta-analysis, systematic review, topical acne treatment, triple-combination gel

## Abstract

Acne vulgaris (AV) is a common dermatological condition that ranges from mild comedones to severe inflammatory nodules and scarring. Effective management is essential for improving patients’ quality of life. The recent FDA approval of IDP-126 (Cabtreo™), a novel triple-combination gel, meets these needs by combining clindamycin phosphate, benzoyl peroxide, and adapalene into a single formulation. This systematic review and meta-analysis aimed to assess the efficacy, safety, and impact of IDP-126 on quality of life in managing AV. The study’s goal was to provide clinicians with the necessary information to consider including this medication in acne treatment plans. A total of 281 articles were manually screened, and three studies (n = 388) met the inclusion criteria. Standardized mean differences were used to quantify lesion reductions, while pooled ORs assessed adverse events. Additional references were reviewed to address potential gaps in the reported outcomes. IDP-126 showed significant reductions in inflammatory and non-inflammatory lesions compared to vehicle treatments and demonstrated favorable numbers needed to treat compared to other topical therapies. It was associated with application site pain and erythema. Notable improvements in quality of life were observed across various domains related to acne severity. IDP-126 is an effective treatment for AV, offering substantial clinical benefits and improving quality of life. While it is associated with higher rates of certain adverse effects, its overall efficacy supports its inclusion in treatment regimens, provided that its safety profile is carefully managed to optimize patient outcomes.

## Introduction and background

Acne vulgaris (AV) is the eighth most common cutaneous disease, with clinical manifestations ranging from occasional non-inflammatory comedones to chronic inflammatory nodules that can result in residual scarring and hyperpigmentation [1-4]. Treatment regimens for acne should be tailored to the patient’s specific presentation, with options including topical, oral, and laser/light therapies. While current studies highlight oral isotretinoin as the most effective treatment for AV, combination topical regimens have been shown to be more effective than oral medications or topical monotherapies [5,6]. These topical treatments include antibiotics, benzoyl peroxide, and retinoids. When topical treatments are preferred over oral options, combining all three categories of therapies enhances treatment outcomes [5,6]. However, adherence challenges may arise when patients are prescribed complex multi-medication regimens [5,6]. To address this, the FDA recently approved the first triple therapy gel, IDP-126 (Cabtreo™), combining clindamycin phosphate (1.2%), benzoyl peroxide (3.1%), and adapalene (0.15%) [7]. This formulation is believed to improve efficacy, tolerability, and patient adherence [8]. Additionally, the concurrent application of all three compounds may reduce the risk of antibiotic resistance, further enhancing the effectiveness of AV treatment [9].

Several studies have outlined the efficacy and safety of IDP-126 [8,10,11]. However, no comprehensive analysis has consolidated all available data on this emerging treatment option. Therefore, the objective of this study was to conduct a thorough evaluation of the efficacy and safety profile of IDP-126 in the management of AV. Additionally, this review aims to examine the impact of IDP-126 on patient quality of life. With its recent market release, this paper serves as a resource for clinicians considering the use of this triple therapy, aiding them in making informed, evidence-based decisions regarding the management of AV.

## Review

Protocol and registration

This systematic review and meta-analysis was conducted in accordance with the Preferred Reporting Items for Systematic reviews and Meta-Analyses (PRISMA) guidelines. The protocol for this review was registered in the PROSPERO database under registration number CRD42024530651.

Eligibility criteria

Eligibility criteria were established using the Population, Intervention, Control, and Outcome (PICO) model. The population included patients of any age diagnosed with AV. The intervention was once-daily IDP-126 gel (Cabtreo™), which combines clindamycin phosphate (1.2%), benzoyl peroxide (3.1%), and adapalene (0.15%). The control intervention was the IDP-126 vehicle gel. The primary outcomes were the absolute and percent changes in inflammatory and non-inflammatory lesions, as well as adverse events.

All prospective comparative studies meeting these criteria were included. Additional inclusion requirements included publication before March 2024, results available in English, and full-text availability. Studies such as reviews, those without results, case reports, case series, editorials, notes, letters, comments, and conference abstracts or posters were excluded, as they did not provide the comprehensive results necessary for this comparative analysis.

Selection process

Two investigators independently conducted screening, data extraction, and quality evaluation based on the inclusion and exclusion criteria between May 1, 2024, and May 20, 2024. The search strategy utilized the terms: "(IDP-126 OR IDP126 OR CABTREO) OR (clindamycin phosphate AND benzoyl peroxide AND adapalene)." Five electronic databases were searched, including PubMed, Scopus, EMBASE, ClinicalTrials.gov, and Cochrane. The investigators ensured consistency in identifying and excluding studies, independently extracted the data, and compared their findings for accuracy. Any discrepancies were discussed and reevaluated to reach a consensus.

Study selection

**Figure 1:**
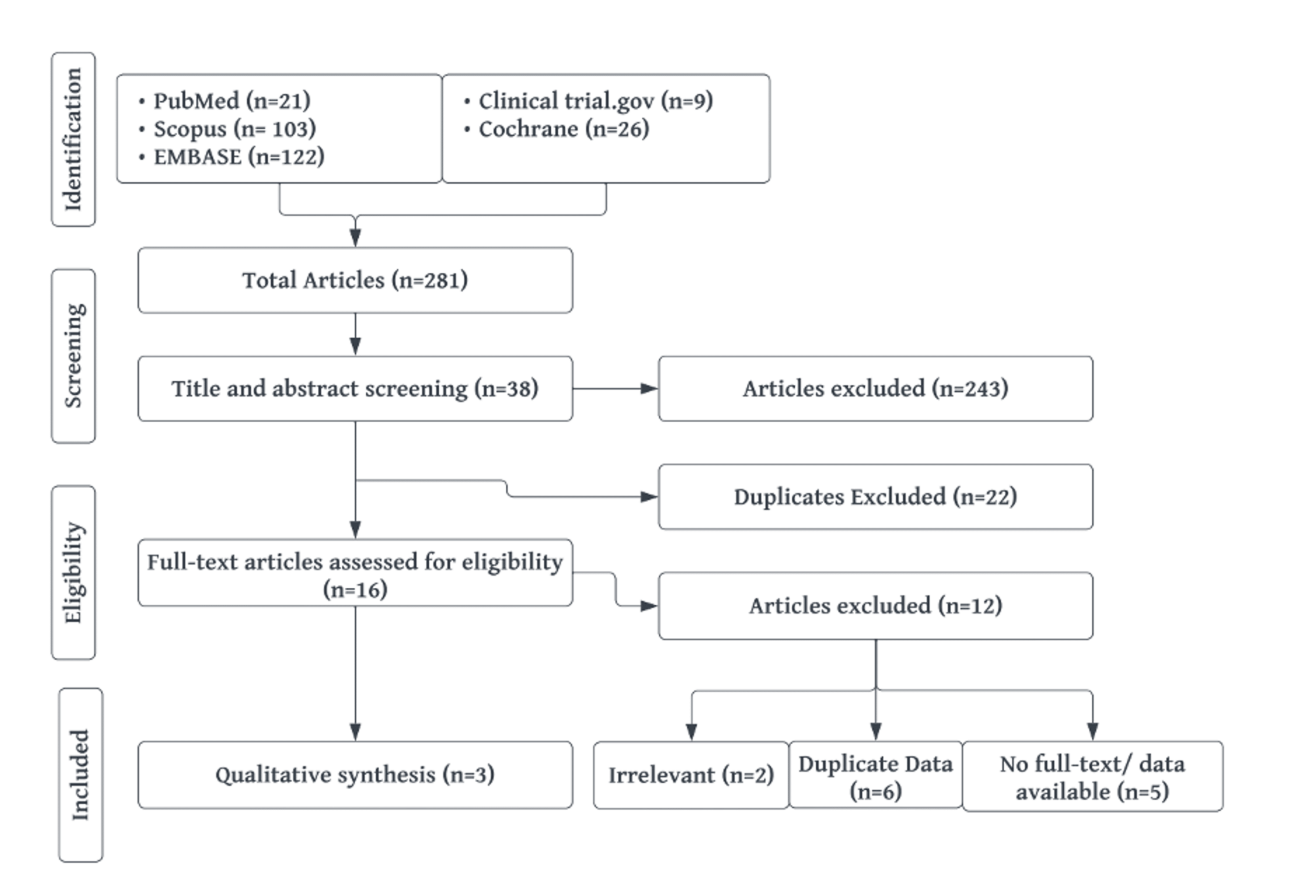
Flow diagram of the screening and selection process

A total of 281 articles were manually screened for titles and abstracts based on the inclusion criteria, leading to the exclusion of 243 articles. Of the remaining 38, 23 duplicate articles were removed, and 15 were selected for full-text review. After eliminating data duplicates, irrelevant articles, and studies with no reported data, three studies were ultimately included in the analysis (Figure 1). The lead investigator’s contact information for the study without reported data could not be located. The majority of data were extracted from ClinicalTrials.gov, with supplemental information gathered from the full-text report on PubMed. Participant characteristics and demographics are presented in Table 1 and Table 2. Additionally, three more studies were identified through manual searching. As these were the only additional studies discussing this medication, they were included to ensure a comprehensive review of this topical treatment. In total, six studies were reviewed for this analysis. Further details on the characteristics of the included studies and their limitations can be found in Appendix A.

**Table 1:** Characteristics and demographics of the included studies’ participants * Severity was determined using the Evaluator’s Global Severity Score grading system [8,10,11].

Study	Median age (years)	Age range (years)	Female (count)	Male (count)	Participants with moderate acne (count)*	Participants with severe acne (count)*
Study 1: NCT03170388 (n = 294)	17	11-47	189	111	251	43
Study 2: NCT04214639 (n = 183)	17	10-44	106	77	165	18
Study 3: NCT04214652 (n = 180)	18	10-48	106	74	166	14

**Table 2:** Number of participants by race in the included studies [8,10,11]

Study	Black/African American	Asian	Native Hawaiian/Pacific Islander	American Indian/Alaska Native	White	2+ races
Study 1: NCT03170388 (n = 294)	50	27	2	3	193	19
Study 2: NCT04214639 (n = 183)	37	17	2	0	120	7
Study 3: NCT04214652 (n = 180)	17	9	0	0	147	7

Data analysis

**Figure 2:**
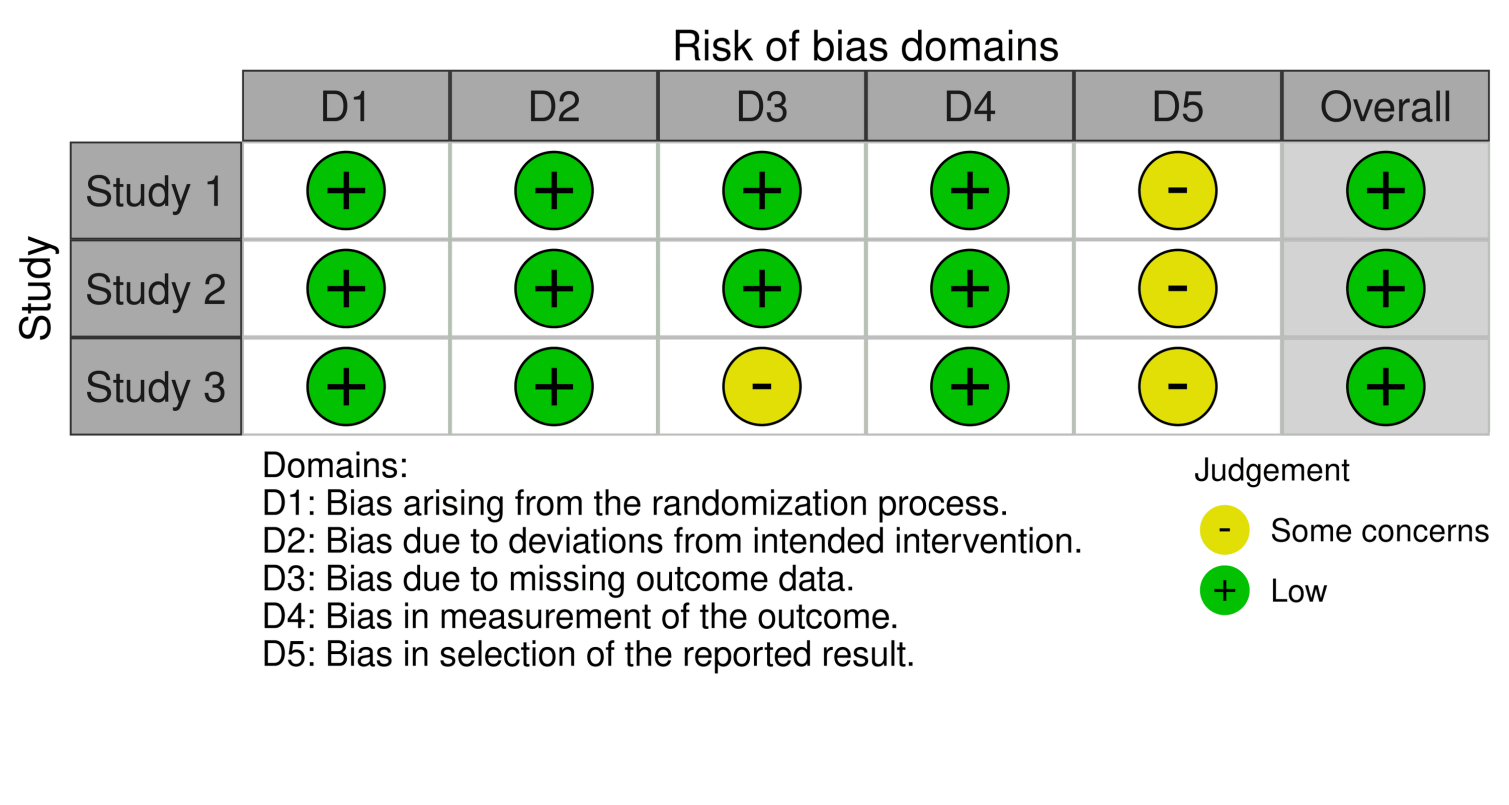
Revised Cochrane risk of bias assessment across the five key domains for the three included studies Study 1 [8], Study 2 [10], and Study 3 [11] were evaluated using the Cochrane risk of bias assessment tool [12] and graphed with the online robvis software [13].

All data analyses were conducted using R statistical software (version 4.4.1; www.r-package.org). Meta-analyses were performed using either fixed or random effects models via the “meta” package to calculate pooled estimates of standardized mean differences (SMDs) with corresponding 95% CIs for assessing mean and percent reductions in inflammatory and non-inflammatory lesion counts relative to IDP status. Additionally, pooled ORs were calculated to evaluate the association between IDP status and binary outcomes, including adverse events, application site pain, and erythema. Since heterogeneity (I² value) did not exceed 50% with statistical significance, the fixed effects model was used for the meta-analyses. Publication bias was assessed by inspecting funnel plot asymmetry for each meta-analysis and conducting Egger’s regression test. All p-values were two-sided, with p < 0.05 considered statistically significant. The risk of bias was evaluated using the revised Cochrane risk of bias tool for randomized trials and visualized using the online robvis software (Figure 2) [12,13].

Efficacy

**Figure 3:**
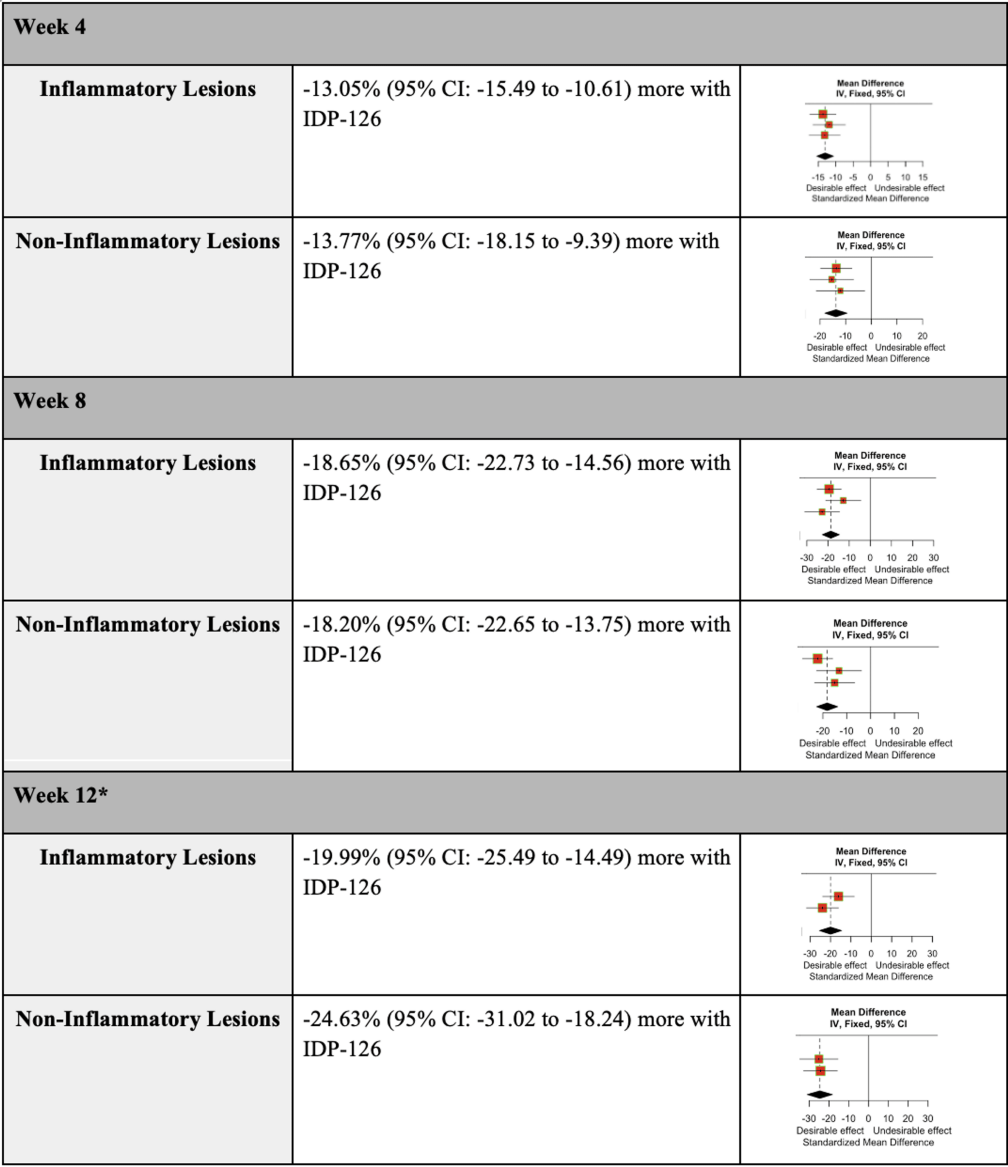
Weighted mean difference in the percent change of inflammatory and non-inflammatory lesions at weeks 4, 8, and 12 in participants using IDP-126 gel compared to vehicle gel * One of the studies did not report the percent change in inflammatory and non-inflammatory lesions for week 12 [8,10,11].

The efficacy of IDP-126 was evaluated by assessing the weighted mean count and percent difference in inflammatory and non-inflammatory lesions. At 12 weeks, IDP-126 users showed a reduction of 8.22 inflammatory lesions (95% CI: -9.87 to -6.58) and 13.05 non-inflammatory lesions (95% CI: -15.49 to -10.61) compared to vehicle gel users. Additionally, IDP-126 users exhibited a greater percent decrease in both inflammatory and non-inflammatory lesions at weeks 4, 8, and 12 (Figure 3).

**Figure 4:**
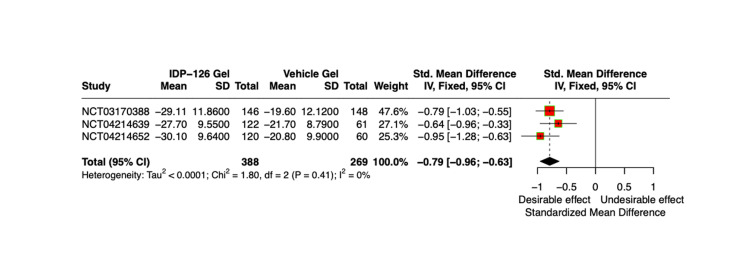
Comparison of weighted mean inflammatory lesion counts relative to IDP-126 application after 12 weeks, using SMDs pooled estimates with a 95% CI SMD, standardized mean difference [8,10,11]

**Figure 5:**
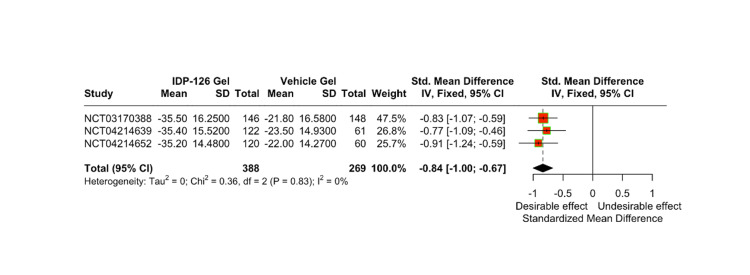
Comparison of weighted mean non-inflammatory lesion counts relative to IDP-126 application after 12 weeks, using SMDs pooled estimates with a 95% CI SMD, standardized mean difference [8,10,11]

The mean count and percent difference in inflammatory and non-inflammatory lesions relative to IDP-126 application were evaluated using pooled SMDSs with a 95% CI. After 12 weeks, IDP-126 users consistently demonstrated greater reductions in both inflammatory and non-inflammatory lesion counts compared to vehicle gel users (Figure 4, Figure 5). At weeks 4 and 8, the SMDs for the percentage of inflammatory lesions in the vehicle group were 0.54 and 0.71 units smaller, respectively, compared to the IDP-126 group. Similarly, at weeks 4 and 8, the SMDs for the percentage of non-inflammatory lesions in the vehicle group were 0.49 and 0.63 units smaller, respectively, compared to the IDP-126 group.

An additional study, not included in the analysis, compared the efficacy of topical acne medications using the number needed to treat (NNT) metric. The study found that IDP-126 had one of the most favorable NNT values, with scores of four and five across two different trials. The only other medication with a comparable NNT was Adapalene 0.3%/BPO 2.5% gel, which had an NNT of five [14].

Side effects

The pooled ORs were calculated to assess the association between IDP-126 status and binary outcomes, including treatment-emergent adverse events (TEAEs), application site pain, and erythema. The odds of experiencing any TEAE were 3.52 times higher in IDP-126 users compared to vehicle gel users, with 30.2% of IDP-126 users reporting a TEAE. Application site pain was the most common side effect, with the odds of experiencing pain being 12.57 times higher in IDP-126 users compared to vehicle gel users, and 11.3% of IDP-126 users reported application site pain. The second most common side effect was erythema, observed in 3.4% of IDP-126 users, with the odds of erythema being 5.98 times higher in IDP-126 users compared to vehicle gel users. Additional side effects, although reported by only a few participants, included sickle cell anemia crisis (n = 1), enteritis (n = 1), application site dryness (n = 9), and viral upper respiratory tract infection (n = 10). An additional study, not included in the analysis, evaluated the safety profile of IDP-126 and found that it may cause allergic sensitization, with two out of 206 participants (1.0%) experiencing a reaction suggestive of allergic sensitization. This study also found that IDP-126 is moderately irritating, but less so than BPO 2.5%/adapalene 0.3% gel [15].

Impact on quality of life

Although not presented in the included studies, a post hoc exploratory analysis involving 309 participants was conducted to assess the effect of IDP-126 on quality of life. The analysis revealed that the topical medication significantly improved quality of life across all domains of the Acne-Specific Quality of Life Questionnaire, including role-emotional, self-perception, acne symptoms, and role-social. Furthermore, the improvement in acne severity was found to significantly influence the enhancements in quality of life [16].

Discussion

IDP-126 (Cabtreo™), a novel triple-combination gel containing clindamycin phosphate, benzoyl peroxide, and adapalene, has demonstrated significant promise in treating AV. Clinical trials have shown its efficacy in notably reducing both inflammatory and non-inflammatory lesions, with consistent improvements observed at 4, 8, and 12 weeks. The favorable NNT further supports its superior efficacy compared to other topical treatments. Additionally, IDP-126 has a positive impact on patients’ quality of life, enhancing emotional well-being and self-perception, thereby making it a promising therapeutic option for acne management through its combination of multiple active agents.

However, healthcare professionals should be mindful of potential adverse effects associated with IDP-126, including application site pain and erythema. These side effects, which are also common with other acne medications, should be considered when assessing patient adherence to treatment. Furthermore, while a generic version is not yet available, clinicians should be aware of the medication’s cost, although discounts for cash-paying patients and coverage options for those with commercial insurance may help reduce financial barriers. By carefully considering these factors, healthcare providers can optimize treatment outcomes and enhance patient satisfaction.

Limitations

The individual studies had several limitations, including inter-observer bias in scoring acne severity using the Evaluator's Global Severity Score (EGSS), temporal bias due to the studies being limited to 12 weeks, and sampling and selection bias, as the demographics used may not fully represent real-world practice populations [8,10,11]. The current meta-analysis also has its limitations, including a small sample size. Given that IDP-126 is a new drug, only three clinical trials were available for analysis, all of which were conducted by the same drug company. Despite these limitations, this analysis is valuable in pooling all available data on the product. Additionally, there was a lack of comparisons with other acne treatments, as the study primarily focused on evaluating the efficacy and side effects of IDP-126 against the vehicle gel. Future studies should assess the efficacy of IDP-126 in comparison to other available acne therapies. Furthermore, one study lacked a reported mean percent difference in inflammatory and non-inflammatory lesions for the 12-week assessment, which is an important data gap.

## Conclusions

Overall, IDP-126 presents a promising treatment option for AV, demonstrating strong clinical outcomes alongside significant improvements in patients’ quality of life. However, its side effects must be carefully managed to optimize therapeutic benefits, necessitating thoughtful patient monitoring. Additionally, factors such as affordability and long-term safety could influence patient adherence, highlighting the need for future longitudinal research to fully assess the long-term impact of IDP-126 on acne management.

## Appendices

Appendix A

**Table 3:** Studies included in analysis * Quality rating based on modified Oxford Centre for Evidence-based Medicine [8,10,11].

Title	ClinicalTrials.gov identifier	Type of study	Participants (count)	Rating of quality*
A Phase 2, Multicenter, Randomized, Double-Blind, Vehicle Controlled, Parallel-Group, Clinical Study Comparing the Efficacy and Safety of IDP-126 Gel in the Treatment of Acne Vulgaris	NCT03170388	Phase II, double-blind, multicenter, randomized, 12-week study Parallel Assignment	741 total; 146 with IDP-126 gel; 148 with vehicle gel	1
A Phase 3, Multicenter, Randomized, Double-Blind, Vehicle Controlled, Parallel-Group, Clinical Study Comparing the Efficacy and Safety of IDP-126 Gel in the Treatment of Acne Vulgaris	NCT04214639	Phase III, double-blind, multicenter, randomized, 12-week study Parallel Assignment	183 total; 122 with IDP-126 gel; 61 with vehicle gel	1
A Phase 3, Multicenter, Randomized, Double-Blind, Vehicle Controlled, Parallel-Group, Clinical Study Comparing the Efficacy and Safety of IDP-126 Gel in the Treatment of Acne Vulgaris.	NCT04214652	Phase III, double-blind, multicenter, randomized, 12-week study	180 total; 120 with IDP-126 gel; 60 with vehicle gel	1
